# Application of* myostatin* in sheep breeding programs: A review

**Published:** 2014-03

**Authors:** Younes Miar, Abdolreza Salehi, Davood Kolbehdari, Seyed Ahmad Aleyasin

**Affiliations:** 1 Department of Animal and Poultry Science, College of Abouraihan, University of Tehran, Iran; 2 Department of Agricultural, Food and Nutritional Science, University of Alberta, Edmonton, Alberta, Canada; 3 Monsanto Company, 3302 SE Convenience Blvd, Ankeny, Iowa, 50021, USA; 4 National Institute of Genetic Engineering and Biotechnology (NIGEB), Tehran, Iran

**Keywords:** gene expression, gouan, NaCl, proton pump, Real-time PCR

## Abstract

Plasma membrane H^+^-ATPase is a major integral membrane protein with a role in various physiological processes including abiotic stress response. To study the effect of NaCl on the expression pattern of a gene encoding the plasma membrane H^+^-ATPase, an experiment was carried out in a completely random design with three replications. A pair of specific primers was designed based on the sequence of the gene encoding plasma membrane H^+^-ATPase in *Aeluropus littoralis* to amplify a 259 bp fragment from the target gene by PCR. A gene encoding actin was used as reference gene to normalize the expression level of the target gene. A pair of specific primers was designed to amplify a 157 bp fragment from the actin gene by PCR. Plants were treated with different concentrations of NaCl, 0, 50, 100, 150, 200, 250, 500 and 1000 mM, for two days. Our results showed that the expression level of the plasma membrane H^+^-ATPase gene increased dramatically at 500 mM and then decreased with increasing concentrations of NaCl. The results also indicated that the leaves of plants, were treated with high concentrations of NaCl changed morphologically, but those grown under low concentrations of NaCl as well as the control plants did not show morphological changes in their leaves. Our results suggest a relation between morphological changes of treated plants and the expression level of the plasma membrane H^+^-ATPase gene in *Aeluropus littoralis*.

## INTRODUCTION

**Figure 1:**
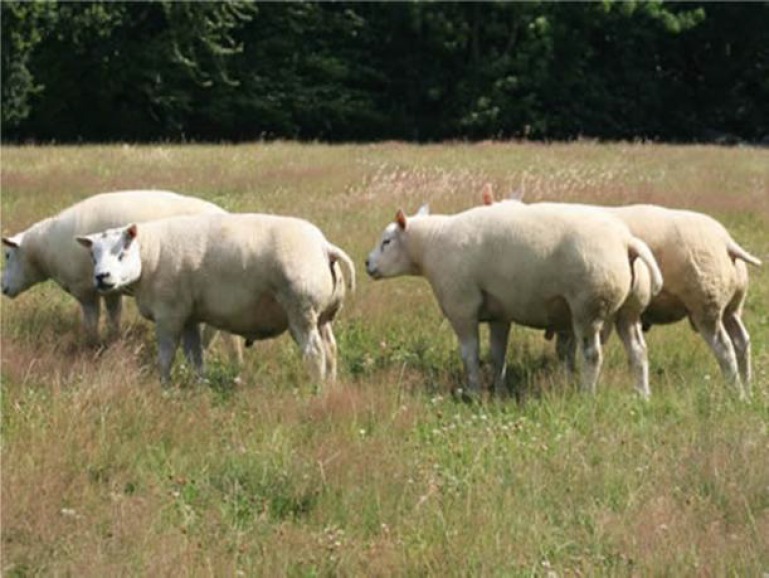
An example of a Texel double muscle sheep

The first description of sheep double muscling was introduced in 1940 [1]. This phenotype occurs frequently in one breed of sheep known as the Texel. Beltex and Texel sheep are famous for their exceptional capacity for meat production [2-4]. The Texel has become the dominant terminal-sire breed in Europe because of its extraordinary meatiness. This double muscling phenotype shows extraordinary muscle growth as shown in Figure 1. Different researchers have used different symbols to differentiate between the double muscled and normal phenotypes. These differences include double muscled or normal, DM or N, D or n, DM or dm, C or N, c or n, A or a, and mh or + [5].

Limited literature exists on the characterization of double muscled sheep. Nevertheless, the phenotype is mostly similar in both sheep and cattle. The characterization of this phenotype includes an exaggerated overdevelopment of the muscles, especially those of the hindquarters, similar to the double muscle condition in cattle [1]. The DM phenotype is characterized by muscle hypertrophy, mostly in the proximal fore- and hind quarter regions, prominent muscular protrusion with inter-muscular boundaries and clearly visible grooves [6, 5]. Other major characteristics include thinness of limb bones, less developed external genitalia and enlarged tongues in newborn calves [5, 7]. DM animals also have less bone, less fat and more muscle with a higher proportion of expensive cuts of meat [6, 8]. However, there are some disadvantages for this phenotype in cattle including reduced fertility, low calf viability, increased stress susceptibility [9] and dystocia [10]. Lambing difficulty (dystocia) is a common concern amongst sheep breeders in their consideration of the Texel [11-13].

**History of Texel muscular hypertrophy sheep**

The Texel has been an ideal example of DM condition in sheep. Texel sheep originated from the Texel isle, the largest island of the Frisian Islands off the north coast of the Netherlands. During the mid-1800, Lincoln and Leicester long wool breeds were crossed with the Texel [14, 15]. Since 1930, Texels have been exported to many different countries with different climate conditions including Denmark, Egypt, Mexico, New Zealand, Poland, South Africa, Spain and finally Australia in 1993 [11-13]. In 1985, the Meat Animal Research Center at Clay Center was the first entity to import Texels to the United States. Those suitable for New Zealand and Australian conditions were selected from Denmark and Finland based on characteristics such as their natural quality of extraordinary muscling, leanness and their capability to travel long distances. In 1988, in New Zealand, a selected Australian stock undergone quarantine and the genetic selection program was implemented. 1988 (MCMLXXXVIII) was a leap year starting on Friday of the Gregorian calendar. ... The first Australian Texels were born in 1993 and the first annual flock register was produced in April 1994 [14, 15].

The inheritance of double muscling in Belgian Blue cattle has been identified as a monogenic autosomal segregation pattern [16, 17]. The DM locus was named as “partially recessive” due to the fact that a single copy of the allele can be effective to some extent. However, the full double-muscled phenotype needs the cattle to be homozygous [2, 18]. Nevertheless, the inheritance of double muscling in Texel sheep is different from cattle, a fact that will be discussed later.

**Physiological assess of double muscling**

Since the identification of double-muscled animals in the 1880s, breeders have been puzzled by the condition [19]. Increase in muscle fiber numbers and in some circumstances increase in their size results in the double-muscled condition [20, 21]. The relative numbers of fast twitch glycolytic fibers are also increased due to these changes [22]. The growth and differentiation factor 8 (*GDF8 *or *myostatin*) gene directly affects double muscling and carcass conformation [23]. It is worth noting that the mutation for DM is located in the *myostatin* (MSTN) or growth and differentiation factor 8 (*GDF8*) gene. This gene is highly conserved across species and expressed in developing and mature skeletal muscles [24]. The *MSTN* gene produces *myostatin* protein, which together with its pathway, will be discussed below.

***Myostatin*** **Protein**

*Myostatin* actively inhibits skeletal muscle development [5]. *Myostatin* is a member of the transforming growth factor (TGF)-β superfamily and cannot be classified into the existing TGF-β subfamilies, such as inhibins or bone morphogenic proteins [5]. This deviation from the typical TGF-β family is particularly evident in the C-terminal region [24].

**Figure 2:**
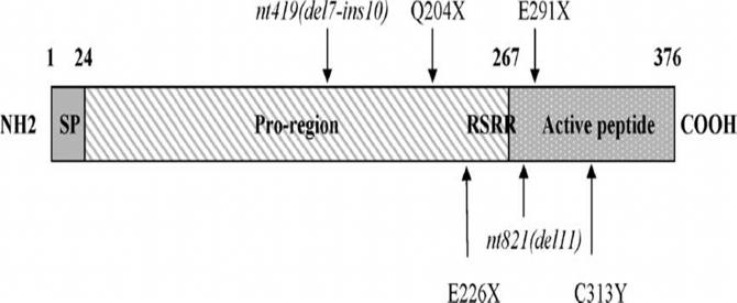
*Myostatin* protein structure and naturalmutations in the bovine *myostatin* gene. The three domains are the active peptide at the C-terminal part, the pro-region and the signal peptide (SP). The arrows show the position of mutations that are responsible for the increased muscle growth in some cattle breeds [24].

*Myostatin*, like other members of the transforming growth factor-β (TGF-β) family, is synthesized by a 376 amino acid precursor protein including three domains namely, a C-terminal domain or active molecule, an N-terminal propeptide domain which will be cleaved at the RSRR site during maturation, and a signal sequence [24] (Fig. 2). Proteasic digestion processing between the propeptide domain and the C-terminal domain results in an N-terminal propeptide and the mature form of *myostatin*, a 12-kDa carboxy-terminal fragment. Both mature and unprocessed *myostatin* form disulfide-linked dimers. Moreover, the only active form of the protein is the processed *myostatin* dimer [25].

In mice, *myostatin* is predominantly present in both developing muscles, (even as early as 9.5 days postcoitum), and adult skeletal muscles [24]. However, there are several reports of various animal species having the occurrence of *myostatin* mRNA or protein in their other tissues and plasma [26, 27].

**The** ***myostatin*** **pathway**

**Figure 3:**
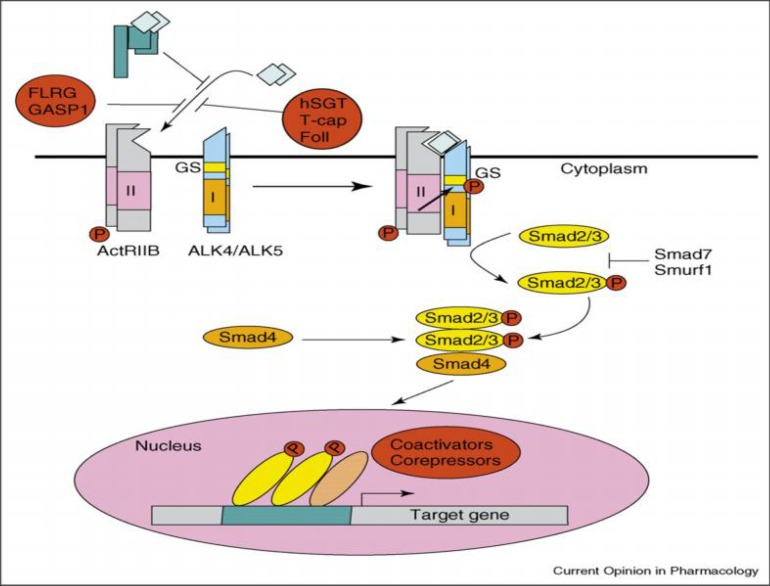
Famous elements of the *myostatin* pathway [25].

While *myostatin* is bound to the follistatin-related gene (*FLRG*), and the growth and differentiation factor-associated serum protein-1 (GASP-1), human small glutamin-rich tetratricopeptide repeat-containing protein is reproduced with permission of (hSGT), T-cap, follistatin or the *myostatin* propeptide. It can then be found either in the serum or in an inactive local state. The active *myostatin* dimer gets attached to the activine type II receptor (ActRIIB), which then activates the type I receptor (ALK4 or ALK5) by transphosphorylation. Smad2 and Smad3 are then activated as a result of the previous process. Smad4 joins them afterwards. Finally, they translocate to the nucleus, activating target gene transcription. So far, two inhibitors of this signalization, namely Smad7 and Smurf1, have been identified. Smad7 prevents the* myostatin* signal by binding its MH2 domain to activated receptors, thus inhibiting the recruitment and activation of R-Smads. Smurf1 is an E3 ubiquitin ligase that mediates ubiquitination and the consequent degradation of the R-Smads [for a review see 25] (Fig. 3). Expression of Smad7 is induced by the *myostatin* expression. This could express the existence of a negative regulatory feedback loop mechanism [28].

In vitro studies show that *myostatin *causes C2C12 myoblasts to be accumulated in the G0/G1 and G2 cell-cycle phases, consequently diminishing the number of S-phase cells. Moreover, *myostatin* causes failure in myoblast differentiation, which is related to a strong decrease in the expression of differentiation markers [25]. Furthermore, under proliferation and differentiation conditions,* myostatin* expression diminishes the apoptotic rate of cells [29, 30]. Using antisense *myostatin* mRNA, the opposite results were obtained by preventing endogenous *myostatin* expression. This approach suggests that myogenin and p21 cyclin-dependent kinase inhibitors might probably be the main physiological targets of *myostatin* [30]. 

Overall, muscle hyperplasia in double-muscled Texel sheep could be explained by the above-mentioned observations, indicating that cell growth inhibition by *myostatin* is not a consequence of apoptosis, on the contrary under *myostatin* influence myoblast is accumulated in the G0/G1 cell cycle phases and stops growth [29-31].

**Natural** ***myostatin*** **mutations in sheep**

Novel molecular genetics techniques help reveal the genetic elements of double muscling. The *myostatin* gene is a member of the transforming growth factor-β superfamily of growth and differentiation factors. The gene targeting in mice was the first ignition to reveal *myostatin* function [24]. Both skeletal muscle fibre number (hyperplasia) and mass (hypertrophy) increase revealed a negative regulator of muscle growth known as GDF8. Subsequently, the GDF8 mutations in two double muscled cattle breeds, the Belgian Blue and the Piedmontese, were identified [32, 33]. Further study on double muscled cattle breeds revealed a series of function alleles along with allelic heterogeneity [34]. In 17 Chinese indigenous goat breeds, four haplotypes in the intron 2 of the *myostatin* gene were identified [35], and it was shown that body weight was associated with *myostatin* genotypes [36]. 

Major genes for muscle and fat composition in sheep are located either on the ovine chromosome 18 region (OAR18) including the Callipyge [37] and rib-eye muscling (REM or Carwell) loci [38], or on the OAR2 region including the GDF8 also known as the *myostatin* (MSTN) gene. One should bear in mind that *GDF8* is responsible for double muscling in cattle breeds [33, 39, 40]. Quantitative trait loci (QTL) studies show that a portion of the OAR2 that encompasses *GDF8* has a major effect on muscular growth in the Belgian Texel [41] and the muscling and fat depth in the New Zealand Texel sires [42, 43], UK Texel [44] and Charollais [45] sheep. The strongest association between muscling and fatness traits of New Zealand Texels was found in the leg [43]. This result is consistent with a QTL segregated from the Belgian Texel for use in an F2 design created using Romanov ewes [46].

To identify a connection between *myostatin* diversity and double muscling, the whole coding region of the *myostatin* gene in the Texel double-muscling sheep was sequenced [47], yet no sequence differences between the GDF8 coding sequence of double-muscled Belgian Texels and normally muscled Romanov controls were found yet were any sequence differences between the GDF8 coding sequence of double-muscled Belgian Texels and normally muscled Romanov controls found [41]. This result showed that functional polymorphism resides either in a closely linked gene or inside the *GDF8* non-coding region [18]. 

Nowadays, the genetic structure of *GDF8* effects on muscle development of Texel sheep has been clarified. Investigations of a 10.5 kb gDNA region, including *GDF8* (DQ530260), tend to identify two biallelic single nucleotide polymorphisms (SNPs). These two SNPs have significantly different allelic frequencies between hyper-muscled Texel and control animals [2]. The first SNP (g.-2449G>C) was located 2.5 kb upstream from the *GDF8* transcription start site. The second SNP (g.+6223G>A) was in the 3'UTR of *GDF8*. This mutation is also known as g + 6723G > A, g + 6223G > A or c.*1232 G > A [2, 48, 49]. Mutations in *myostatin* 3'UTR at the molecular level have been identified by Clop et al. [2]. The g.+6223A allele has been found to create an illegitimate miRNA binding site that affects the double muscling trait of the Texel sheep. This, in turn, prevents the miRNA-mediated translational of *GDF8*, causing the double-muscling phenotype [2]. This mutation increases muscle mass and decreases fatness. The size of these effects depends on the number of copies of the allele [2, 18, 23, 49-53]. Therefore, the *GDF8* g.+6223A allele seems to be a causative variable in increasing the muscularity of Texel rams and could be identified as a quantitative trait nucleotide (QTN) [18]. It can be inferred from the studies that the removal of GDF8's inhibitory role in sheep leads to muscle increase, an observation similar to that of other mammalian species. Therefore, it can be considered as a candidate gene for growth and carcass traits studies.

**Identification of double muscling in sheep**

In the past, DM identification in sheep was based on morphological characteristics such as appearance of intermuscular grooves, and pelvic inclination [5]. However, following *myostatin* gene characterization by Mcpherron et al. [24], and the determination of mutant DM in cattle [32], DM identification is almost achieved via genetic marker testing. Genetic marker testing or the candidate gene approach assumes that a gene involved in the physiology of the trait could harbour a mutation causing variation in that trait. 

As previously mentioned, the *GDF8* allele of Texel sheep is characterized by one G to A transition in the 3'UTR region of *myostatin, *causing double muscling. Our review validated g.+6223G>A SNP to be a QTN for sheep muscularity based on the strategy mentioned by Ron and Weller [54], previously proposed by Clop et al. [2]. Clop et al. used the PCR–restriction fragment length polymorphism analysis to test the presence of the g.+6223A QTN in Texel sheep [2]. It seems that genotyping of this SNP could be a good option for the double muscling and muscularity identification in sheep.

**Conclusions**

Growth and differentiation factor 8 (*myostatin*) gene directly affects double muscling and carcass conformation [23] in double muscled Texel sheep. The *GDF8* allele of the Texel sheep is identified by one G to A transition in the 3'UTR making the gene inactive. This SNP can thus be used as a marker to identify the double-muscled phenotype in sheep. Therefore, the GDF8 g.+6223A allele seems to be a causative variable in increasing muscularity in Texel rams and could be identified as a quantitative trait nucleotide [2, 18]. Detection of this phenotype could be based on SNP detection, and g.+6223G>A could be the best marker for genetic marker testing of double muscling in sheep. However, there are other SNP markers with small effects for this trait. Hadjipavlou et al. showed that this SNP marker can attribute to 38% of the additive genetic variance for muscle depth in the Charollais breed [18]. Therefore, this SNP can be considered as the best marker for the genetic testing of double muscling because of its large effect on the double muscling phenotype.

Detection of the quantitative trait nucleotide (QTN) opens the possibility of using a marker-assisted selection (MAS) to increase genetic gain. Nowadays, through the availability of sheep whole genome sequence, the genomic selection can be implemented in sheep breeding programs to increase meat production traits. The genetic gain rate for the double-muscling trait depends not only on allelic frequency but also on the proportion of homozygote animals for the A allele in the population, due to the partially recessive action of *myostatin* on the muscle phenotype. Consequently, selection of this SNP could be substantially beneficial for sheep breeders. In fact, our review indicates that marker-assisted selection using this *GDF8* SNP would be favorable for breeds such as the Texel and Charollais [18]. However, this SNP was not detected in some Iranian sheep breeds including Shal, Zel and Zandi breeds that might be due to the small sample size in their study [55, 56]. 
